# Supplementary material for: Deciphering in-situ surface reconstruction in two-dimensional CdPS3 nanosheets for efficient biomass hydrogenation
Source: Nat Commun. 2024 Jun 18;15:5174. doi: 10.1038/s41467-024-49510-8 (PMC11189421; doi:10.1038/s41467-024-49510-8)
Supplement: Supplementary file 1 — Supplementary Information [file 41467_2024_49510_MOESM1_ESM.pdf]

Supplementary information

Deciphering *In-Situ* Surface Reconstruction in Two-dimensional $CdPS_3$ Nanosheets for Efficient Biomass Hydrogenation

Marshet Getaye Sendeku[1,2,3,†], Karim Harrath[4,†], Fekadu Tsegaye Dajan[3], Binglan Wu [3,5], Sabir Hussain[6], Ning Gao[3], Xueying Zhan[3], Ying Yang[5], Zhenxing Wang[3], Chen Chen[7], Weiqiang Liu[2], Fengmei Wang[1,3,*], Haohong Duan[7,*], Xiaoming Sun[1,2,*]

[1]State Key Laboratory of Chemical Resource Engineering, College of Chemistry, Beijing University of Chemical Technology, Beijing, 100029, P. R. China.

[2]Ocean Hydrogen Energy R&D Center, Research Institute of Tsinghua University in Shenzhen, Shenzhen 518057, P. R. China.

[3]CAS Key Laboratory of Nanosystem and Hierarchical Fabrication, National Center for Nanoscience and Technology, Beijing 100190, P. R. China.

[4]Department of Chemistry, Southern University of Science and Technology, Shenzhen 518055, P. R. China.

[5]Shaanxi Provincial Key Laboratory of Electroanalytical Chemistry, Key Laboratory of Synthetic and Natural Functional Molecule of the Ministry of Education, College of Chemistry & Materials Science, Northwest University, Xi'an, 710127, P.R. China.

[6]Tyndall National Institute, University College Cork, Lee Maltings Complex, Dyke Parade, Cork T12 R5CP, Ireland.

[7]Department of Chemistry, Tsinghua University, Beijing, 100084, P. R. China.

[†]These authors contributed equally: M. G. Sendeku and K. Harrath

[*]E-mail: wangfm@buct.edu.cn; hhduan@mail.tsinghua.edu.cn; sunxm@mail.buct.edu.cn

Supplementary Note I

All chemicals and reagents used in this work were received and utilized without further purification. The PBS electrolyte used in this study was directly purchased from ThermoFisher Biochemical Products (Beijing) Co., Ltd, and it was stored at room temperature. The pH of PBS in this study was 9.2 $\pm$ 0.01.

A facile solvothermal method was utilized to synthesize CdS nanoparticles. In detail, commercial powders of thioacetamide (C_2H_5NS, 0.31g) and cadmium nitrate ($Cd(NO_3)_2$, 0.34g) were added to 35 mL isopropanol. The mixture was then sonicated for 10 min, transferred to Teflon–lined stainless steel autoclave, and heated at 150 °C for 12 h to obtain CdS. The resulting product was washed with isopropanol and ethanol, drop-cast on carbon cloth to obtain CdS/CC, and dried overnight in a hot air oven at 60 °C.

The synthesis of $MnCo_2O_{4.5}$ anode is realized via a two-step process. Firstly, the $Co(OH)_2$ precursor was directly synthesized on the nickel foam (NF) substrate through hydrothermal method. Typically, 1.45 g of cobalt nitrate hexahydrate ($Co(NO_3)_2.6H_2O$), 1.5 g urea (CH_4N_2O), and 0.32 g ammonium fluoride (NH_4F) were dissolved in 40 ml of water. The mixture was sonicated for 10 minutes and transferred to the Teflon-lined stainless steel autoclave. A piece of NF substrate (4 x 2 cm^2) was immersed in the autoclave and heated at 120 °C for 6 h to grow $Co(OH)_2$ nanowire. Next, Mn was electrochemically deposited on the as-obtained precursor *via* cathodic deposition process at a constant current of – 20mA for 20 min. The plating electrolyte solution contains 0.1M $Mn(NO_3)_2$. Finally, the as-obtained electrode was annealed at 400 °C for 3h to yield $MnCo_2O_{4.5}$/NF.

The control samples of In_2S_3 and $CdPSe_3$ nanosheets were synthesized *via* solvothermal and space confined chemical vapour conversion methods, respectively. The synthesis protocol for $CdPSe_3$ is almost the same to $CdPS_3$. For the growth of $CdPSe_3$, a powder mixture containing P and Se (1:4, 0.75g) was placed in the front zone, and the as-prepared CdS/CC precursor was kept at the back zone. After pumping the tube with Ar gas for several minutes, the front and back zones were simultaneously heated to 300 and 450 °C, respectively, within 20 min under 100 sccm Ar gas flow. The reaction lasted 60 min, and the final temperature of the front and back zone was 330 and 450 °C, respectively.

Supplementary Note II

Structural characterizations of $MnCo_2O_{4.5}$/NF electrocatalyst. The $MnCo_2O_{4.5}$/NF was also characterized through the XPS analysis. The XPS survey spectrum of the $MnCo_2O_{4.5}$ sample clearly indicates the presence of Mn, Co, and O elements (Supplementary Fig. 32a). The high-resolution XPS of Co 2*p* can be deconvoluted into a pair of peaks (Co $2p_{3/2}$ and 2 $p_{1/2}$) that corresponds to the Co^{3+} (780.7 and 795.7 eV) and Co^{2+} (783.5 and 797.5 eV) along with two satellite peaks at 787.8 and 803 eV. This result suggests the co-existence of two oxidation states of cobalt in the as-prepared $MnCo_2O_{4.5}$ (Supplementary Fig. 32b). Similarly, the high-resolution XPS spectrum of Mn (Supplementary Fig. 32c) can be deconvoluted into a pair peak for Mn^{3+} (644.9 and 655.2eV) and Mn^{2+}(642.2 and 653.6 eV), corresponding to Mn 2 $p_{3/2}$ and Mn 2 $p_{1/2}$. The O 1*s* spectrum exhibit two distinct peaks at binding energies of 530 and 531.5 eV, which belong to the M-O (Co/Mn-O) and hydroxyl species, respectively, on the surface of $MnCo_2O_{4.5}$/NF (Supplementary Fig. 32d).

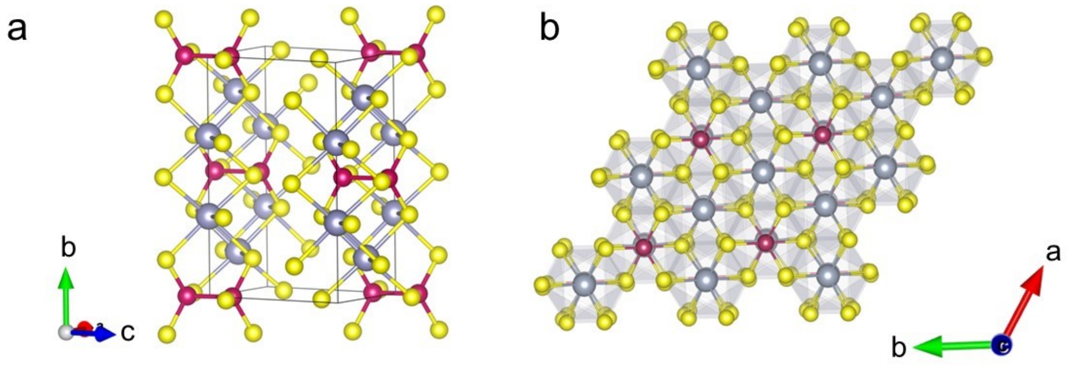

Supplementary Fig. 1| Crystal structure of monoclinic phase $CdPS_3$. a, Unit cell, and **b,** Top view from *c*-direction.

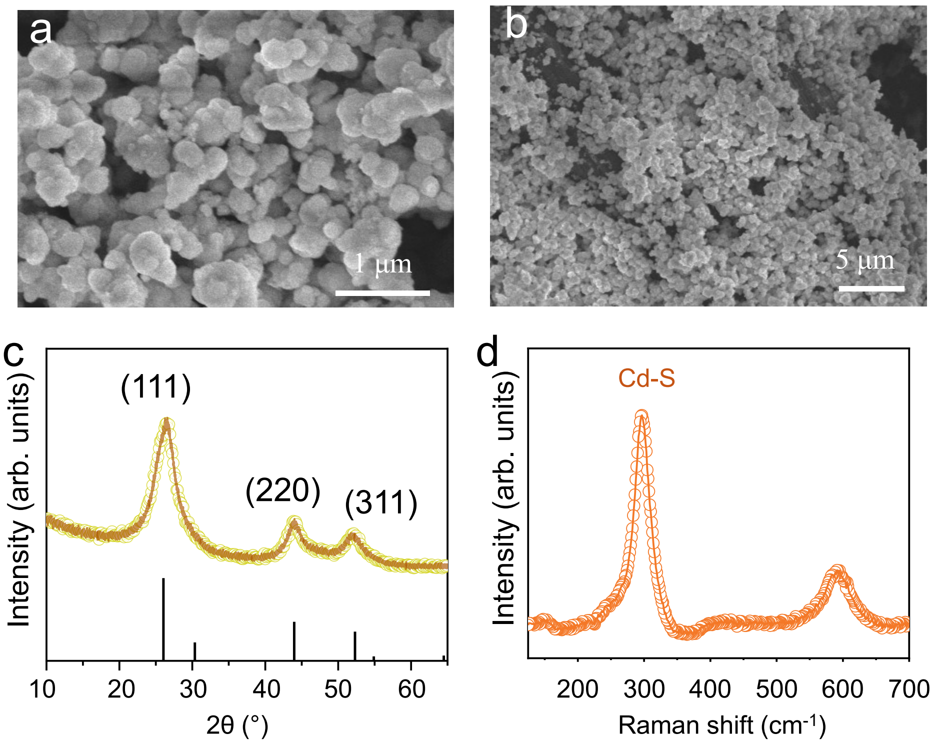

Supplementary Fig. 2| Morphological and structural characterization of the CdS precursor. a,b, SEM images under different magnifications. **c,** XRD pattern of as-synthesized CdS. All XRD peaks are indexed to the cubic phase CdS (PDF#42-1411). **d,** Raman spectrum of CdS precursor.

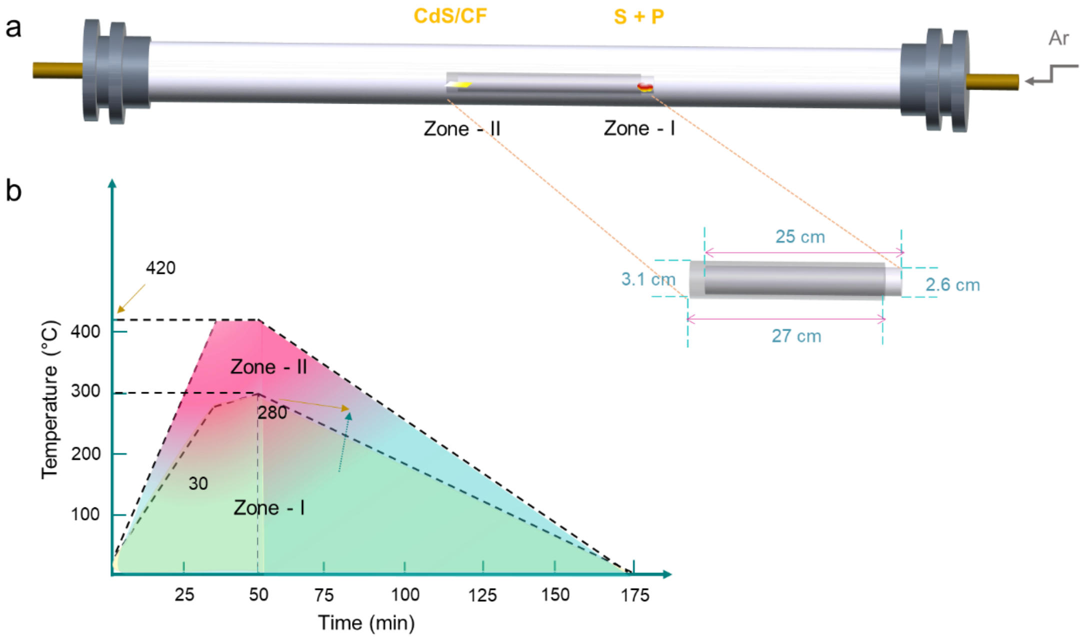

Supplementary Fig. 3| Schematic illustration of the space-confined chemical vapor

conversion strategy for the synthesis of $CdPS_3$ nanosheets on carbon cloth substrate. a, Schematic configuration of the CVD tubes. The custom-designed silica socket tube was used, and the CdS precursor and mixture of P and S were kept at two different ends of the tube. **b,** the temperature profile for the synthesis of $CdPS_3$.

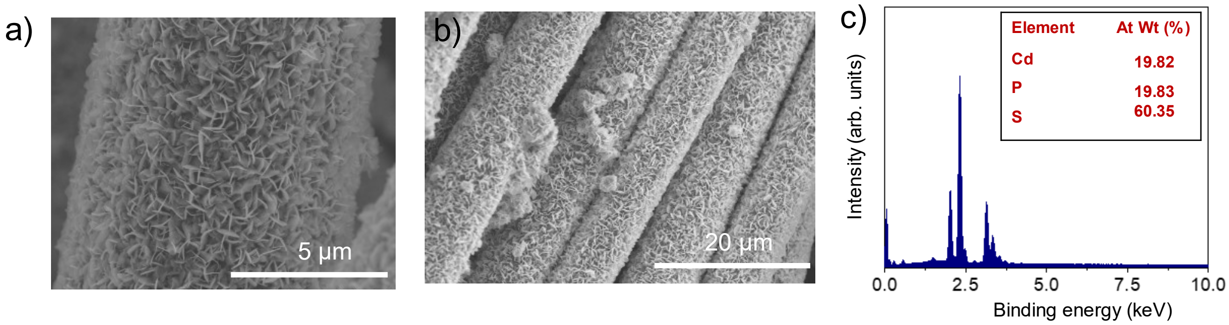

Supplementary Fig. 4| a-b, SEM images of as-synthesized $CdPS_3$ NS on CC. **c,** the corresponding EDX mapping.

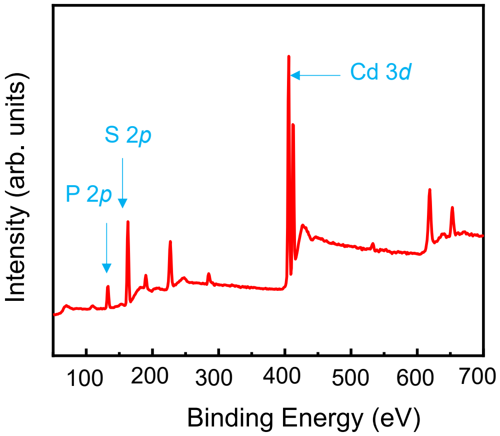

Supplementary Fig. 5| Full XPS spectrum of as-synthesized $CdPS_3$ nanosheet

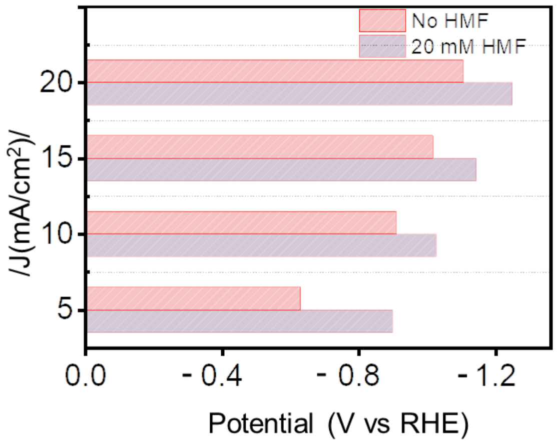

Supplementary Fig. 6| Comparison of the potential at different current densities of $CdPS_3$ electrode tested in the presence and absence of HMF.

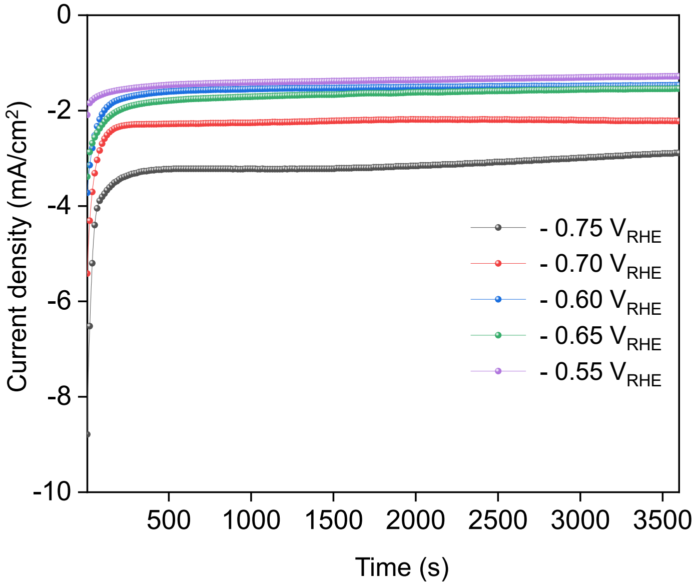

Supplementary Fig. 7| The chronoamperometric test for $CdPS_3$ nanosheet electrode during HMF hydrogenation at different applied potentials (-0.55 to -0.75 V_{RHE}).

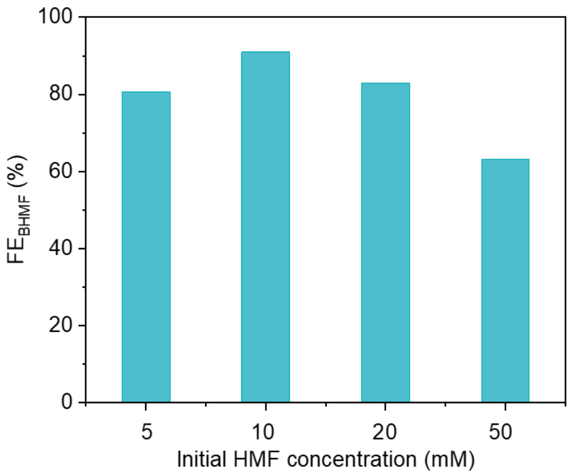

Supplementary Fig. 8| The electrocatalytic HMF hydrogenation performance of $CdPS_3$_25 electrode in 0.1M PBS (pH= 9.2) using different initial concentration of HMF.

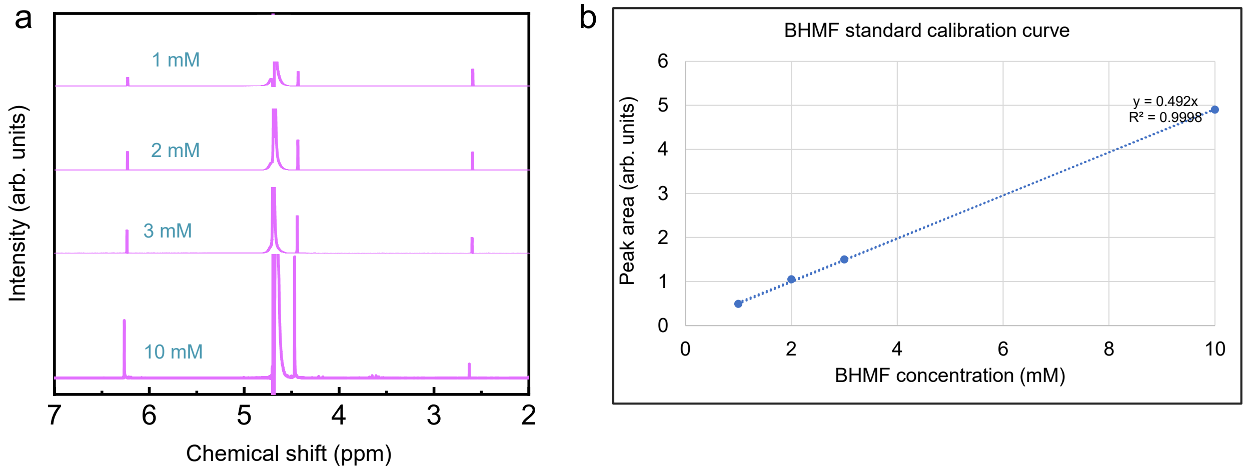

Supplementary Fig. 9| a, The NMR signals of BHMF with different concentration **b,** the corresponding calibration curve.

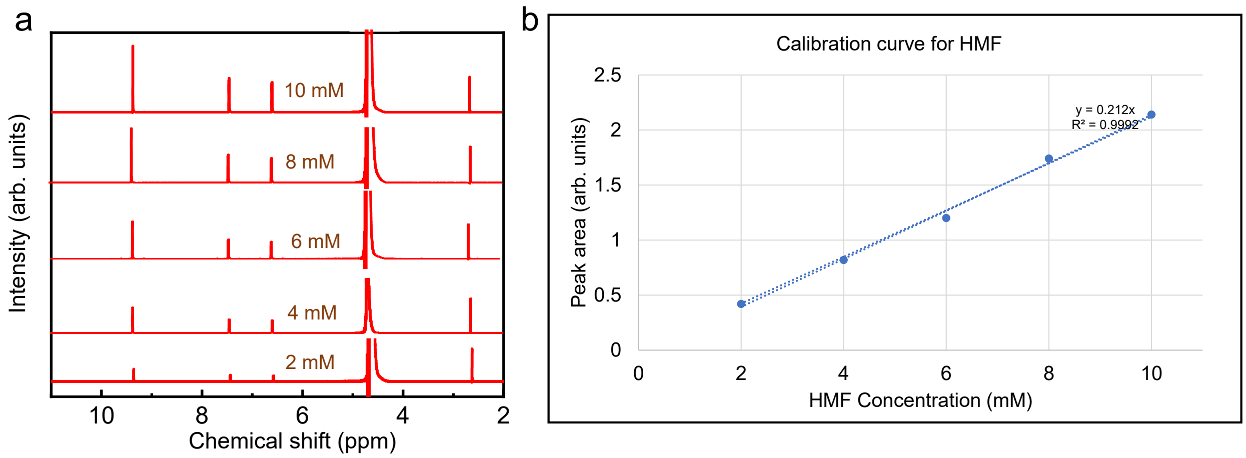

Supplementary Fig. 10| a, The NMR signals of HMF with different concentration **b,** the corresponding calibration curve.

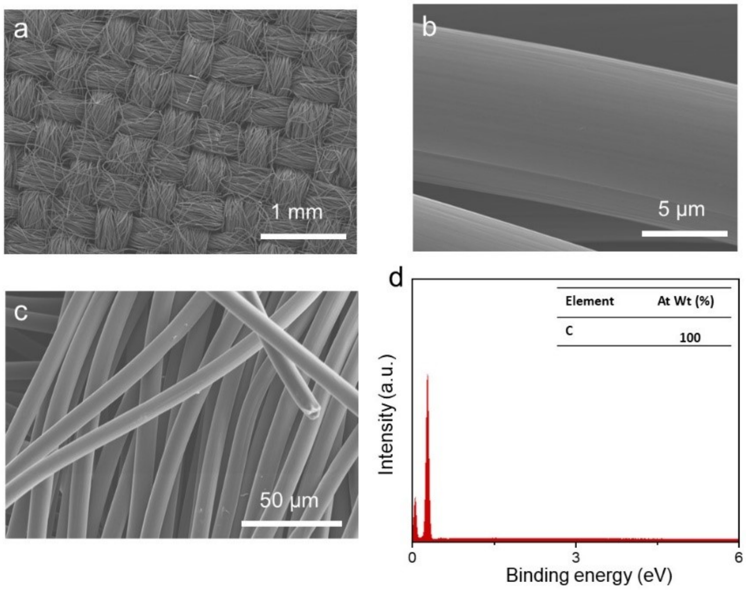

Supplementary Fig. 11| The SEM image (a-c) and corresponding EDX elemental analysis (d) of clean carbon cloth substrate.

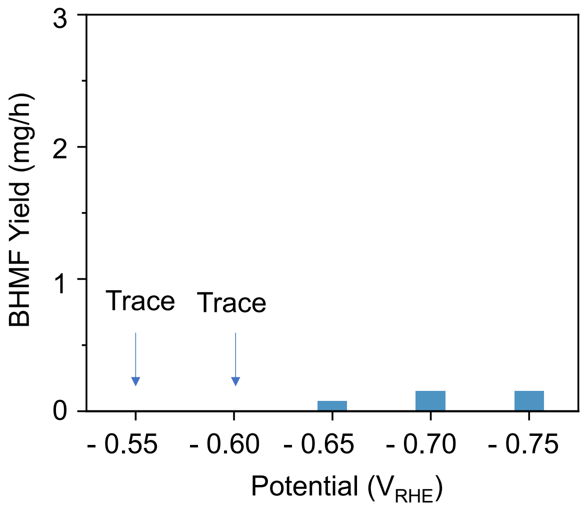

Supplementary Fig. 12| Electrocatalytic performance of carbon cloth substrate toward HMF hydrogenation. The performance of clean carbon cloth substrate was evaluated in 0.1 M PBS (pH = 9.2) electrolyte using 10 mM HMF as the initial substrate concentration.

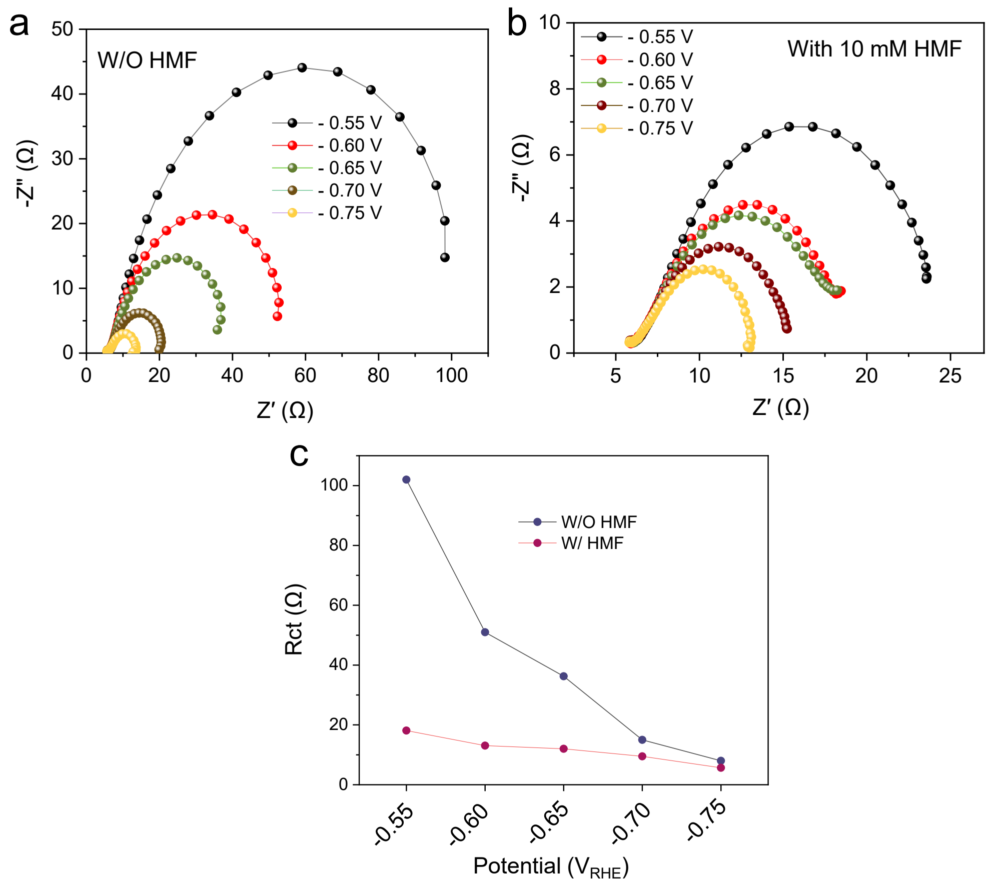

Supplementary Fig. 13| Nyquist plot for $CdPS_3$ electrode in **a,** 0.1 M PBS (pH = 9.2) **b,** 10 mM HMF. **c,** the corresponding charge transfer resistance (Rct) in a and b after fitting.

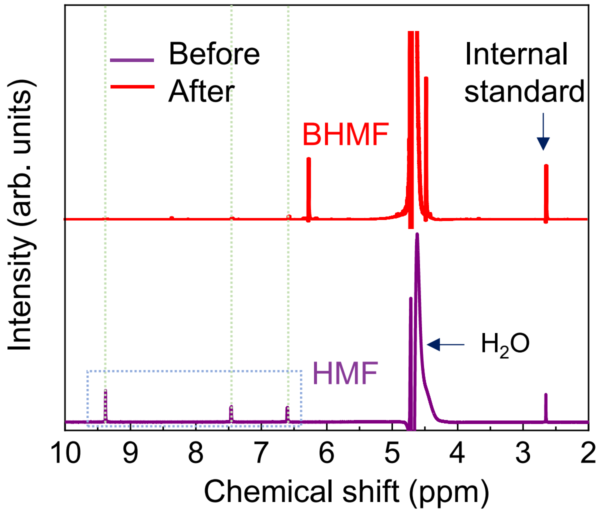

Supplementary Fig. 14| The NMR signal of 10 mM HMF in 0.1 M PBS electrolyte before and after electrolysis at – 0.7 V_{RHE}.

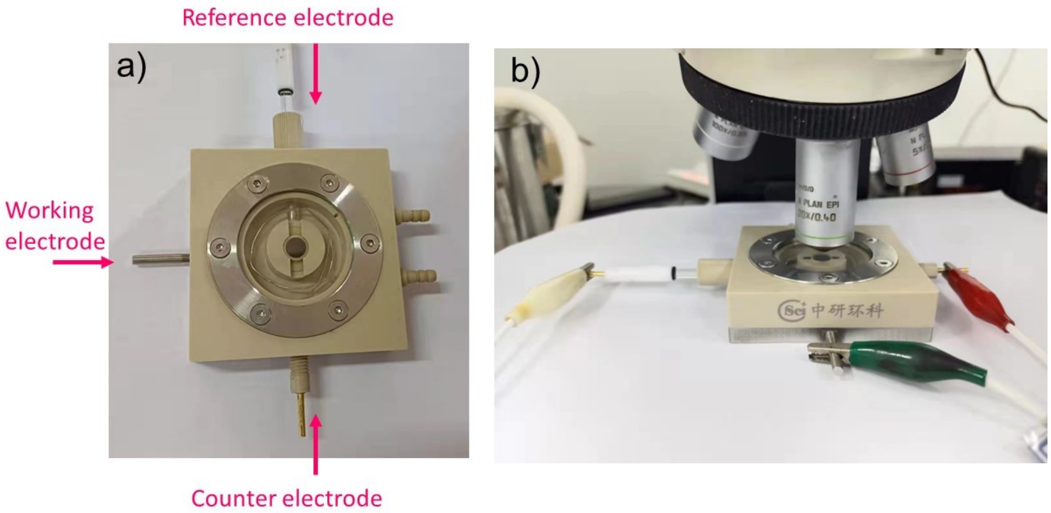

Supplementary Fig. 15| The digital image of electrochemical cell (a) and setup (b) used for *in situ* Raman spectroscopy analysis.

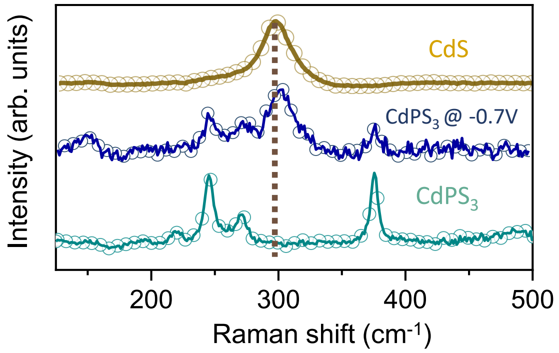

Supplementary Fig. 16| Comparison of Raman spectra of $CdPS_3$, CdS, and $CdPS_3$ under *in situ* Raman testing at – 0.7 V_{RHE}.

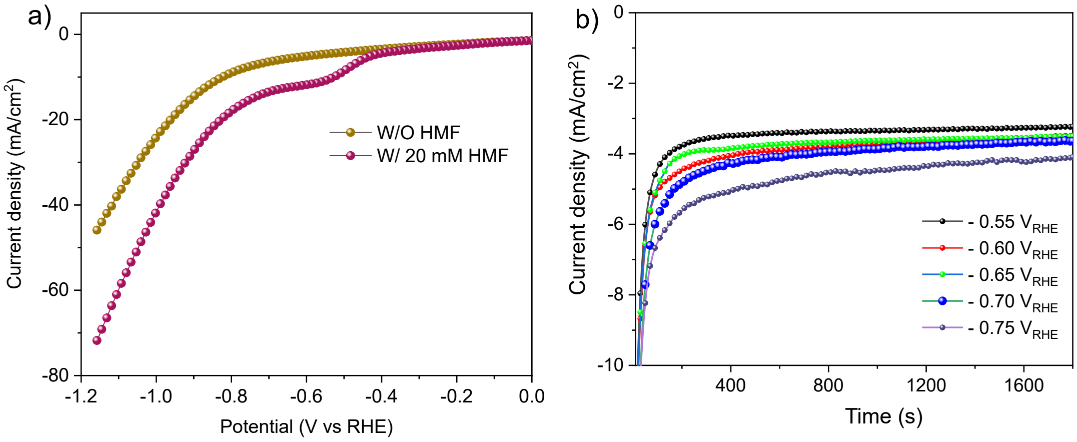

Supplementary Fig. 17| Electrocatalytic activity of CdS sample on carbon cloth (CdS/CC). a, LSV curve (without *iR* correction) of CdS/CC in 0.1 M PBS (pH = 9.2) electrolyte with and without HMF substrate. **b,** The chronoamperometric test for HMF reduction at different applied potentials (-0.55 to -0.75 V_{RHE}).

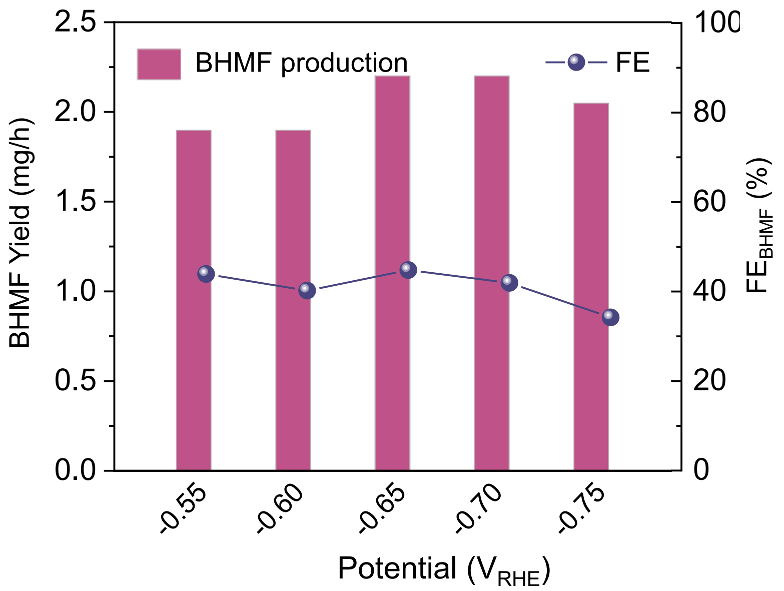

Supplementary Fig. 18| Potential dependent electrocatalytic HMF hydrogenation performance of CdS/CC.

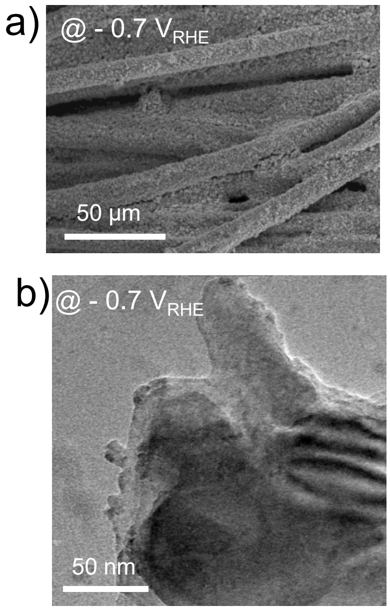

Supplementary Fig. 19| Morphological characterization of the spent over CdPS₃ electrode. a, SEM image. **b,** TEM image after HMF hydrogenation test for 3 hours.

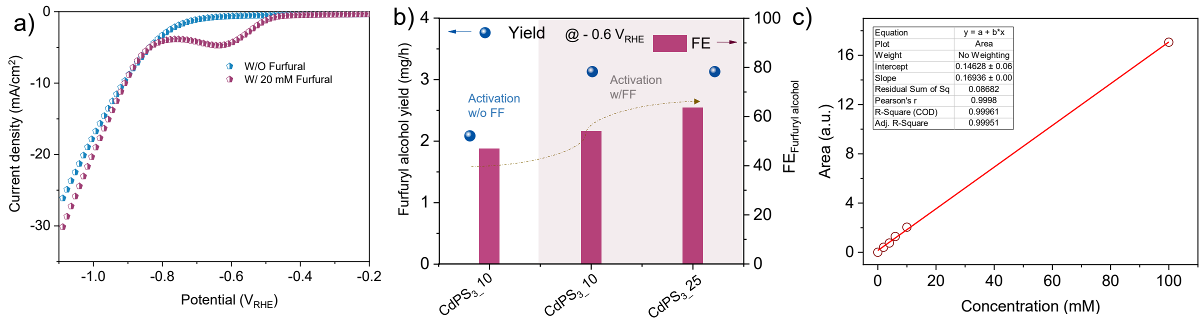

Supplementary Fig. 20| a, LSV curves (without i*R* correction) of $CdPS_3$ electrode tested in 0.1 M PBS (blue) and in 0.1 M PBS + 20 mM furfural (FF). **b,** Comparison of the electrocatalytic furfuryl alcohol synthesis from furfural hydrogenation over $CdPS_3$ electrodes activated in 0.1 M PBS for 10 cycles with those activated in 0.1 M PBS + 10mM HMF for 10 and 25 CV cycles (shaded in light brown color). The performance of these electrodes was evaluated at -0.6 V_{RHE}. **c,** the corresponding calibration curve for furfuryl alcohol quantification.

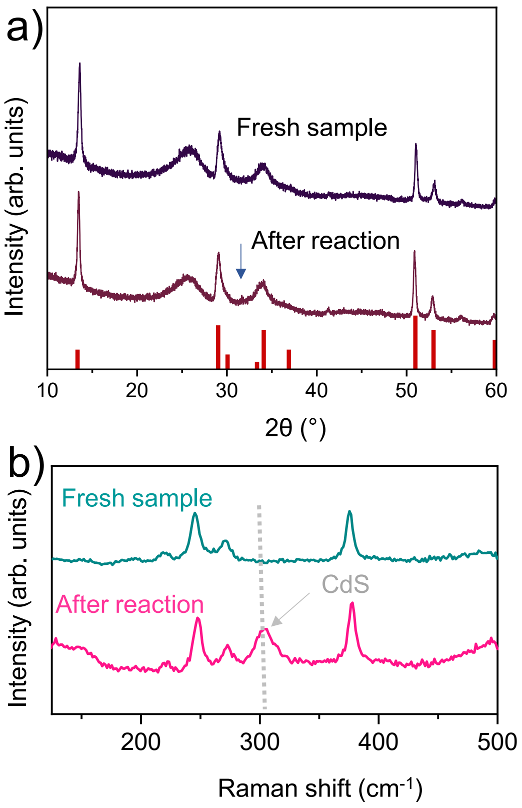

Supplementary Fig. 21| Post structural characterizations of $CdPS_3$ electrode after furfural hydrogenation at - 0.6 V_{RHE}. a, Comparison of XRD diffractogram of fresh and spent over electrode. **b,** The corresponding Raman spectra.

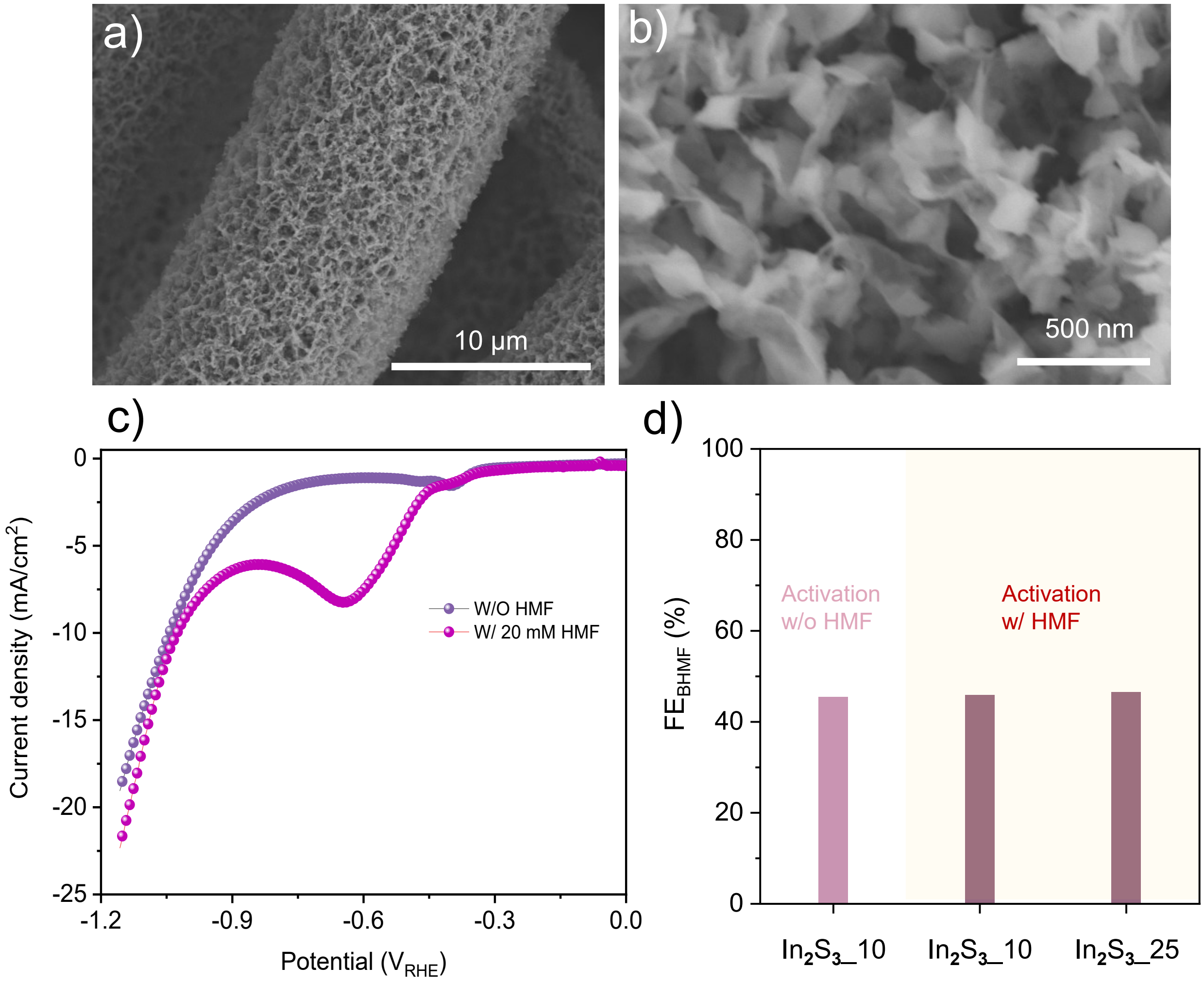

Supplementary Fig. 22| Morphology characterizations and electrocatalytic activity of In_2S_3 nanosheet catalyst for hydrogenation of HMF to BHMF. a-b, SEM images of In_2S_3 nanosheets grown on carbon cloth substrate. **c,** LSV curves (without i*R* correction) of In_2S_3 nanosheet electrode in 0.1 M PBS (pH= 9.2) electrolyte with and without 20 mM HMF. **d,** Comparison of HMF hydrogenation activity of In_2S_3 nanosheet electrodes activated in 0.1 M PBS in the absence of HMF and under 10 and 25 CV activation cycles in the presence of HMF (shaded in light yellow).

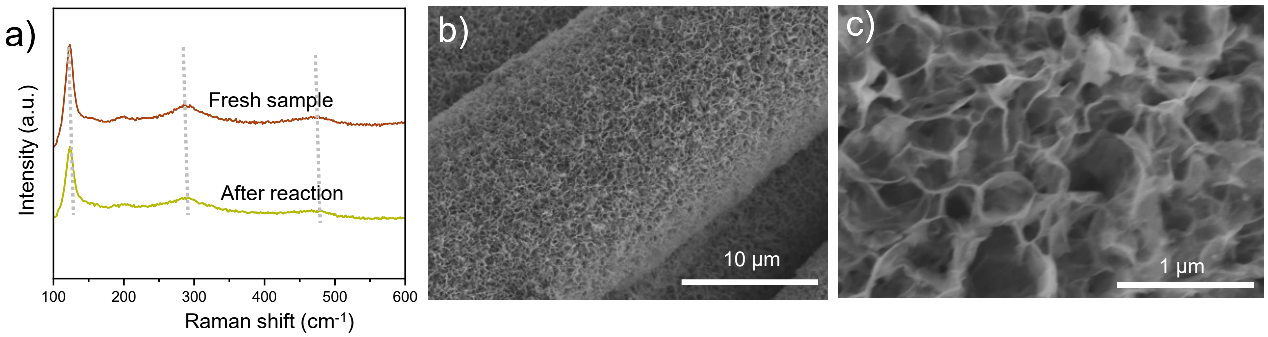

Supplementary Fig. 23| a, comparison of In_2S_3 nanosheets before and after HMF hydrogenation tests using Raman characterizations. **b-c,** The corresponding SEM images of In_2S_3_25 nanosheets after electrocatalytic HMF hydrogenation test.

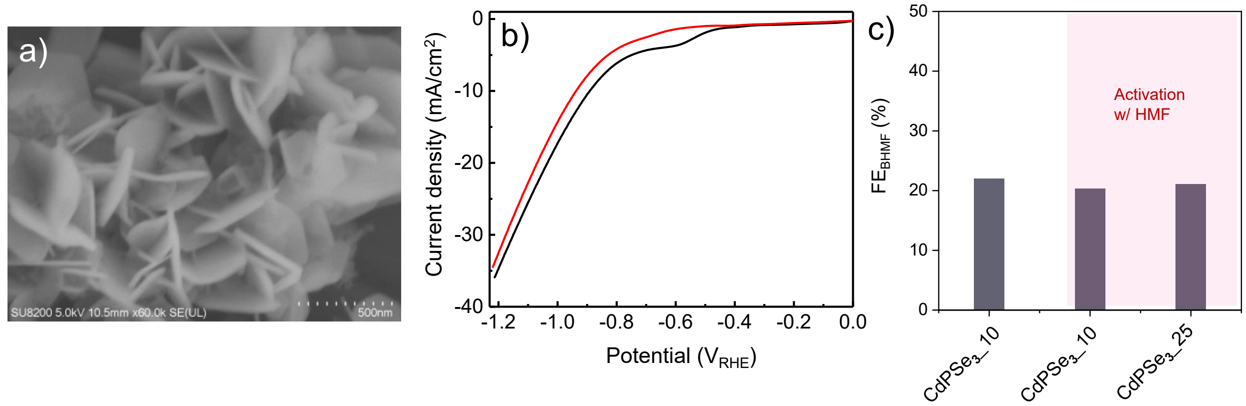

Supplementary Fig. 24| Morphology characterizations and electrocatalytic activity of $CdPSe_3$ nanosheet catalyst for hydrogenation of HMF to BHMF. a, SEM images of $CdPSe_3$ nanosheets grown on carbon cloth substrate. **b,** LSV curves without *iR* correction of $CdPSe_3$ nanosheet electrode directly synthesized on carbon cloth substrate using SCCVC method in 0.1 M PBS (pH= 9.2) electrolyte with (black) and without (red) 20 mM HMF. **c,** Comparison of HMF hydrogenation activity of $CdPSe_3$ nanosheet electrodes activated in only 0.1 M PBS (without HMF) for 10 CV cycles and in the presence of 10mM HMF under 10 and 25 CV activation cycles (shaded with light pink color).

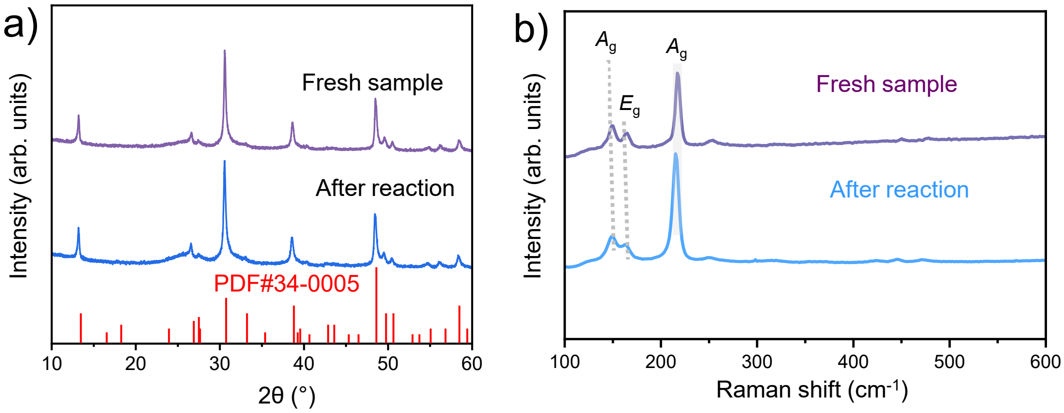

Supplementary Fig. 25| Characterization of $CdPSe_3$_25 nanosheet before and after HMF hydrogenation reaction test. a, XRD patterns. **b,** Raman spectra.

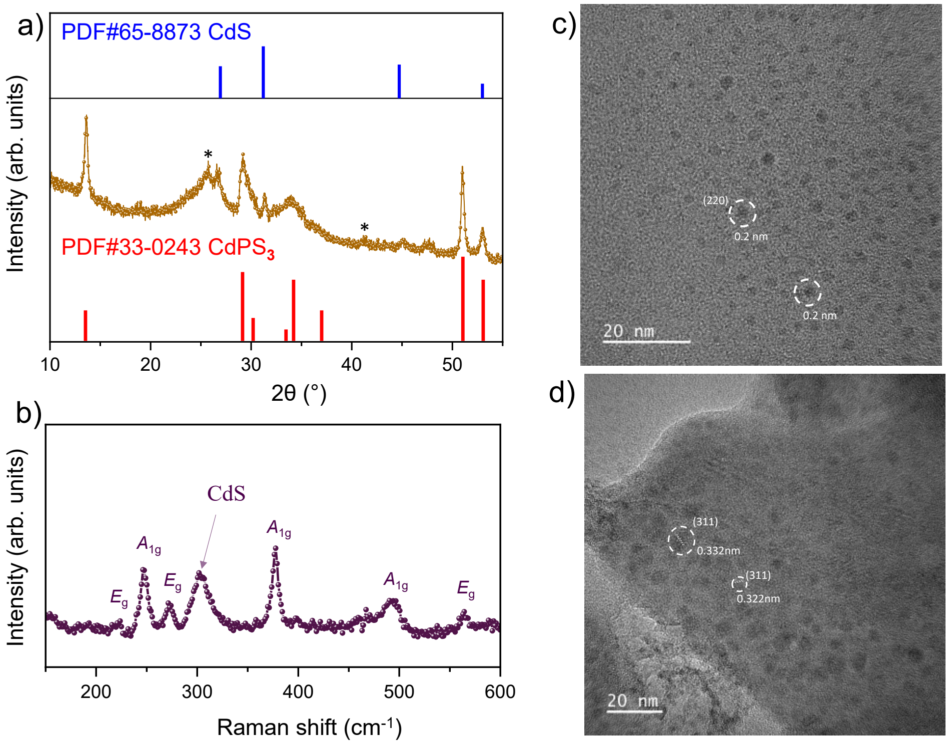

Supplementary Fig. 26| a-b, XRD patterns (a) and Raman spectrum (b) of the as-synthesized $CdPS_3$/CdS_{QD}. **c-d,** High resolution TEM images of the CdS quantum dots decorated on $CdPS_3$ nanosheets. In the XRD pattern, the peaks labelled with * belongs to the carbon cloth substrate.

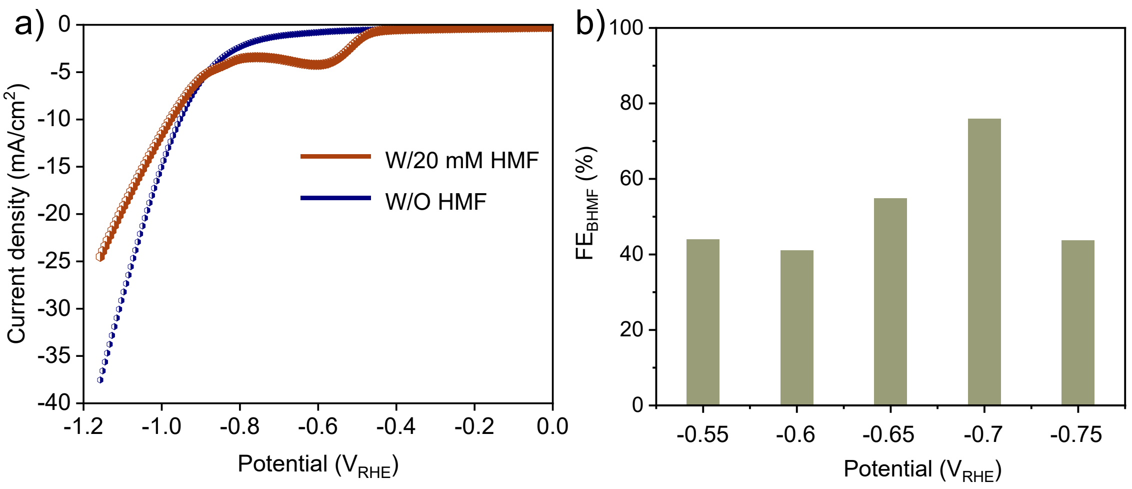

Supplementary Fig. 27| a, LSV curves (without i*R* correction) of $CdPS_3/CdS_{QD}$ electrode tested in 0.1M PBS without and with 20 mM HMF. **b,** Potential dependent electrocatalytic HMF hydrogenation performance of as-synthesized $CdPS_3$-CdS_{QD} electrode.

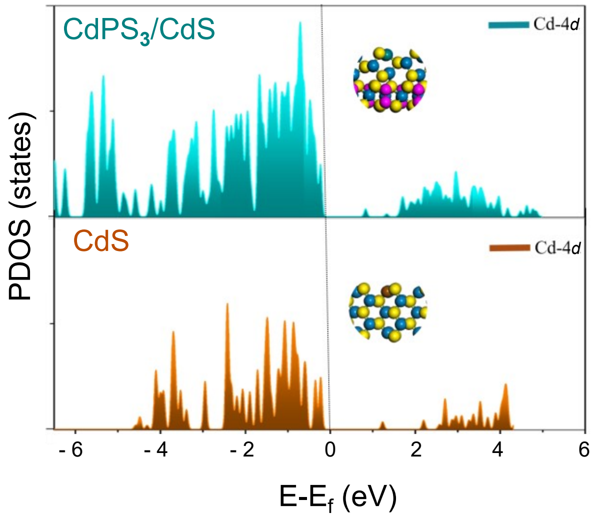

Supplementary Fig. 28| Partial density of state of Cd site on $CdPS_3/CdS$ (upper) interface and CdS (lower) surfaces

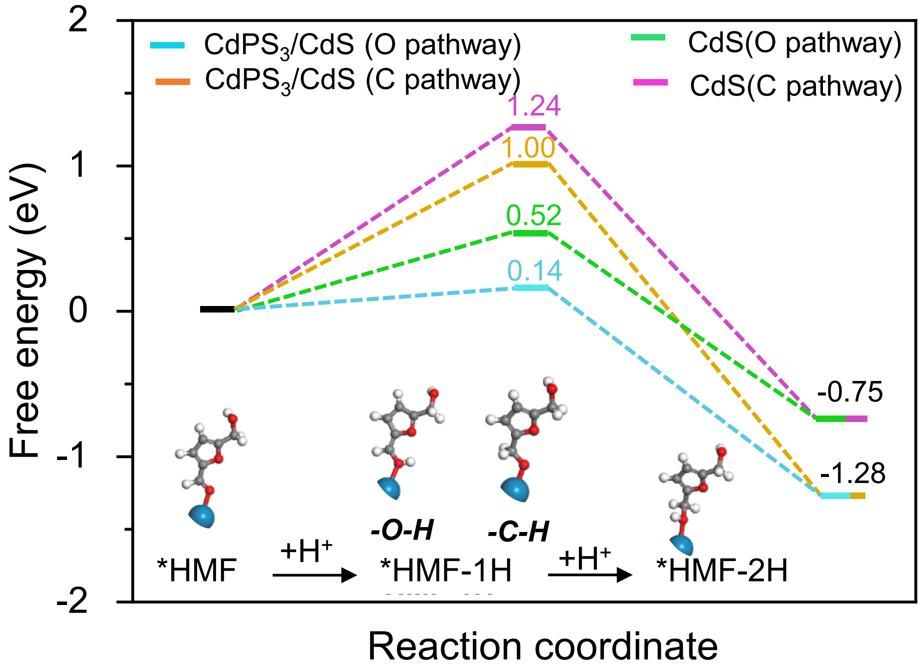

Supplementary Fig. 29| Calculated free energy for HMF hydrogenation on CdS and $CdPS_3$/CdS interface *via* Eley-Rideal mechanism.

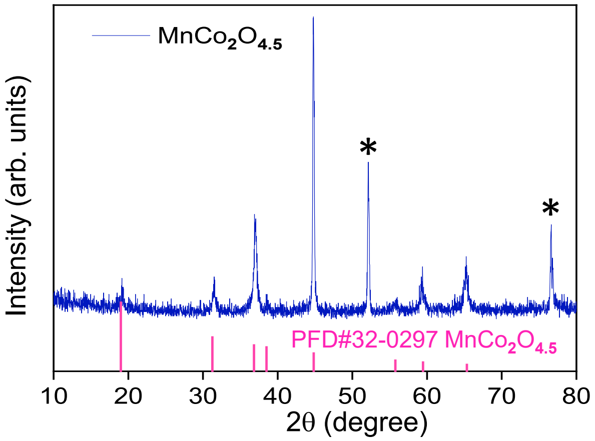

Supplementary Fig. 30| XRD pattern of the as-synthesized $MnCo_2O_{4.5}$ nanorod sample. The peaks labeled as “*” correspond to Ni foam substrate.

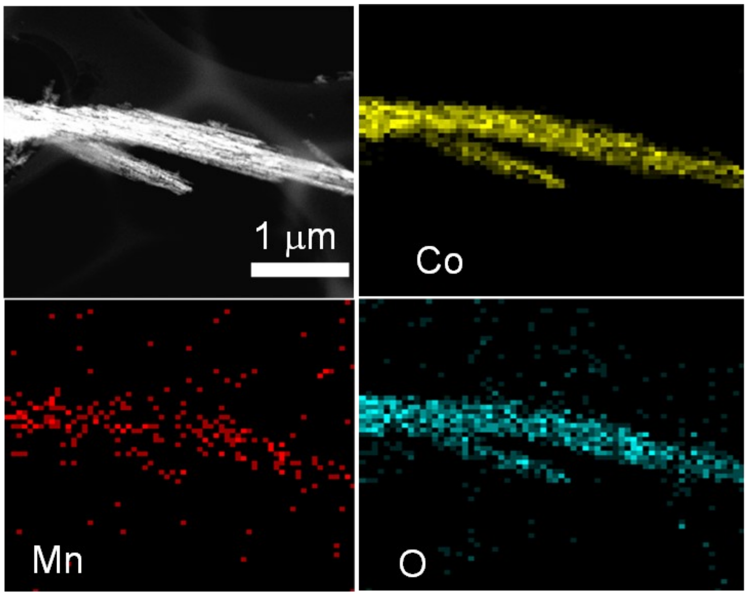

Supplementary Fig. 31| The TEM-EDX mapping for the as-synthesized $MnCo_2O_{4.5}$ nanorod.

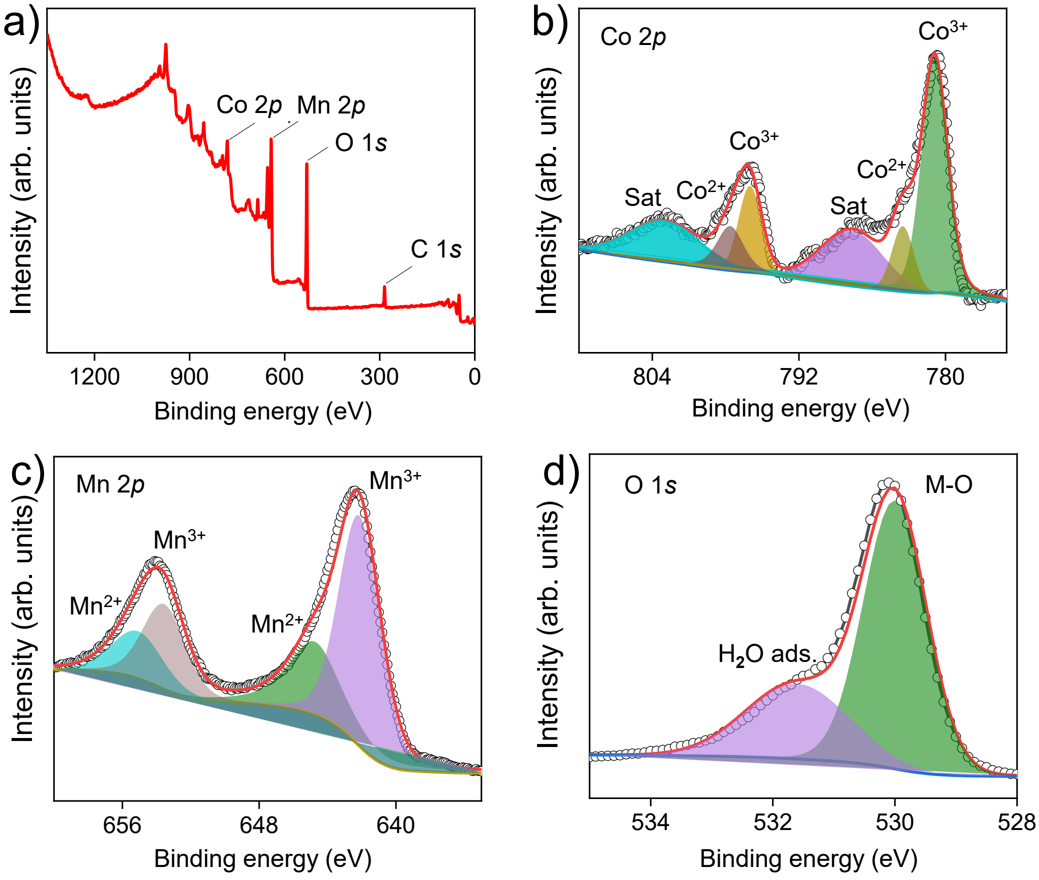

Supplementary Fig. 32| a, Survey XPS spectra of the as-prepared $MnCo_2O_{4.5}$/NF electrocatalyst. **b-d,** High resolution XPS spectra of Co 2*p* (b), Mn 2*p* (c) and O 1*s* (d).

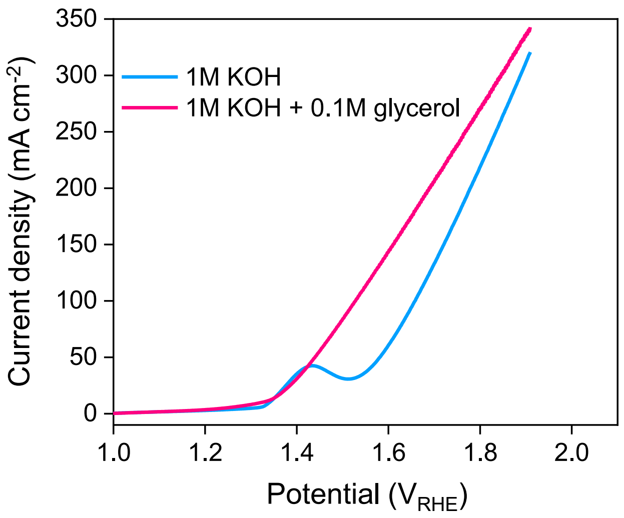

Supplementary Fig. 33| Comparison of LSV curve of $MnCo_2O_{4.5}$/NF in 1M KOH with and without 0.1M glycerol without i*R* correction.

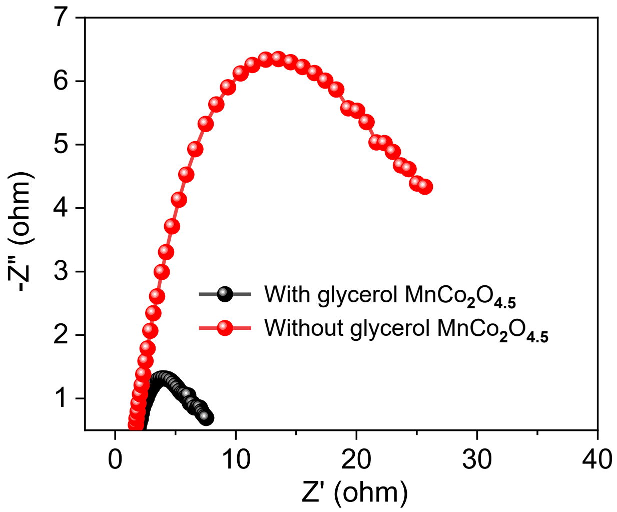

Supplementary Fig. 34| EIS of $MnCo_2O_{4.5}$/NF in the presence of 0.1M glycerol (black) and in 1M KOH (red) at 1.6 V_{RHE}.

Table S1. Comparison of the *in situ* generated $CdPS_3/CdS$ heterostructure electrocatalyst with other reported electrocatalysts

S/N	Electrocatalyst	HMF concentration	Electrolyte (pH)	Potential (V_{RHE})	FE_{BHMF} (%)	Ref
1	AgCu nanoalloys supported by PA	10 mM	0.05 M BBS (9.14)	- 5.4 V [a]	61.8	[1]
2	Ag/Cu GD	50 mM	0.5 M sodium borate buffer (9.2)	− 0.51	85	[2]
3	Ag/C	20 mM	0.5 M sodium borate buffer (9.2)	− 0.56	95	[3]
4	Ag/Cu foam	20 mM	0.5 M sodium borate buffer (9.2)	− 0.51	94	[4]
5	Ru1Cu	20 mM	0.5 M PBS (7)	− 0.3	85.6	[5]
6	RhCu	50 mM	0.5 M Na_2SO_4	-	92.6	[6]
7	OD-Ag	20 mM	0.5 M sodium borate buffer (9.2)	− 0.56	56.2	[7]
8	AgCu	20 mM	0.5 M sodium borate buffer (9.2)	− 0.56	95	[8]
9	MoS_2-DMA	35 mM	0.5 M sodium borate buffer (9.2)	− 0.25	75	[9]
10	$Cu(OH)_2$- ER/CF	5 mM	0.1 M KOH	− 0.15	92.3	[10]
11	Ag/SnO_2	50 mM	0.5 M $KHCO_3$	− 0.62 to -1.12	>95	[11]
12	$CdPS_3/CdS$	10 mM	0.1 M PBS (9.2)	− 0.7	91.3±2.3	**This work**

[a] Two electrode system was utilized and the potential refers to a cell voltage

References

1 Li, H. *et al.* Highly selective electrocatalytic hydrogenation of 5-hydroxymethylfurfural to 2, 5-dihydroxymethylfuran over AgCu nanoalloys. *Int. J. Hydrogen Energy* **47**, 28904-28914 (2022).

2 Sanghez de Luna, G. *et al.* AgCu bimetallic electrocatalysts for the reduction of biomass-derived compounds. *ACS Appl. Mater. Interfaces* **13**, 23675-23688 (2021).

3 Chadderdon, X. H., Chadderdon, D. J., Pfennig, T., Shanks, B. H. & Li, W. Paired electrocatalytic hydrogenation and oxidation of 5-(hydroxymethyl) furfural for efficient production of biomass-derived monomers. *Green Chem.* **21**, 6210-6219 (2019).

4 Sanghez de Luna, G. *et al.* Ag electrodeposited on Cu open-cell foams for the selective electroreduction of 5-hydroxymethylfurfural. *ChemElectroChem* **7**, 1238-1247 (2020).

5 Ji, K. *et al.* Electrocatalytic hydrogenation of 5-hydroxymethylfurfural promoted by a Ru1Cu single-atom alloy catalyst. *Angew. Chem. Int. Ed.* **61**, e202209849 (2022).

6 Zhang, W. *et al.* Rh-dispersed Cu nanowire catalyst for boosting electrocatalytic hydrogenation of 5-hydroxymethylfurfural. *Sci. Bull.* (2023).

7 Liu, H., Lee, T.-H., Chen, Y., Cochran, E. W. & Li, W. Paired electrolysis of 5-(hydroxymethyl) furfural in flow cells with a high-performance oxide-derived silver cathode. *Green Chem.* **23**, 5056-5063 (2021).

8 Zhang, L., Zhang, F., Michel Jr, F. C. & Co, A. C. Efficient electrochemical hydrogenation of 5-hydroxymethylfurfural to 2, 5-bis (hydroxymethyl) furan on Ag-displaced nanotextured Cu catalysts. *ChemElectroChem* **6**, 4739-4749 (2019).

9 Tan, J. *et al.* Interlayer engineering of molybdenum disulfide toward efficient electrocatalytic hydrogenation. *Sci. Bull.* **66**, 1003-1012 (2021).

10 Li, M. *et al.* Facet effect on the reconstructed Cu-catalyzed electrochemical hydrogenation of 5-hydroxymethylfurfural (HMF) towards 2, 5-bis (hydroxymethy) furan (BHMF). *J. Energy Chem.* 84, 101-111 (2023).

11 Guo, X. *et al.* Promoting Electrocatalytic Hydrogenation of 5-Hydroxymethylfurfural over a Cooperative Ag/SnO_2 Catalyst in a Wide Potential Window. *ACS Catal.* **13**, 13528-13539 (2023).
