# Supplementary material for: Deciphering in-situ surface reconstruction in two-dimensional CdPS3 nanosheets for efficient biomass hydrogenation
Source: Nat Commun. 2024 Jun 18;15:5174. doi: 10.1038/s41467-024-49510-8 (PMC11189421; doi:10.1038/s41467-024-49510-8)
Supplement: Supplementary file 3 — Description of Additional Supplementary Files [file 41467_2024_49510_MOESM3_ESM.pdf]

<u>Description of Additional Supplementary Files</u>

File name: Supplementary Data 1

Description: The atomic models of the optimized computational structures used for theoretical calculations in this work.
