# Supplementary material for: Deciphering in-situ surface reconstruction in two-dimensional CdPS3 nanosheets for efficient biomass hydrogenation
Source: Nat Commun. 2024 Jun 18;15:5174. doi: 10.1038/s41467-024-49510-8 (PMC11189421; doi:10.1038/s41467-024-49510-8)
Supplement: Supplementary file 4 — Supplementary Data 1 [file 41467_2024_49510_MOESM4_ESM.pdf]

**The atomic coordinates of the optimized computational models of the $CdPS_3$ structure**

1.0

| | | |
|---|---|---|
| 12.5072002411 | 0.0000000000 | 0.0000000000 |
| 0.0000000000 | 21.6620006561 | 0.0000000000 |
| 0.0000000000 | 0.0000000000 | 29.1695995331 |

Cd P S

16 14 48

Direct

| | | |
|---|---|---|
| 0.249657008 | 0.416034002 | 0.242822006 |
| 0.249651004 | 0.084031997 | 0.242760999 |
| 0.999656951 | 0.166034002 | 0.242822006 |
| 0.999651004 | 0.334031992 | 0.242760999 |
| 0.749656989 | 0.416034002 | 0.242822006 |
| 0.749651042 | 0.084031997 | 0.242760999 |
| 0.499656989 | 0.166034002 | 0.242822006 |
| 0.499651004 | 0.334031992 | 0.242760999 |
| 0.249657008 | 0.916033958 | 0.242822006 |
| 0.249651004 | 0.584031992 | 0.242760999 |
| 0.999656951 | 0.666033980 | 0.242822006 |
| 0.999651004 | 0.834031970 | 0.242760999 |
| 0.749656989 | 0.916033958 | 0.242822006 |
| 0.749651042 | 0.584031992 | 0.242760999 |
| 0.499656989 | 0.666033980 | 0.242822006 |
| 0.499651004 | 0.834031970 | 0.242760999 |
| 0.499936026 | 0.000015000 | 0.204143002 |
| 0.998754990 | 0.000043000 | 0.281698990 |
| 0.249936007 | 0.250014991 | 0.204143002 |
| 0.248754990 | 0.250043013 | 0.281698990 |
| 0.498754990 | 0.000043000 | 0.281698990 |
| 0.749935988 | 0.250014991 | 0.204143002 |
| 0.748754951 | 0.250043013 | 0.281698990 |
| 0.499936026 | 0.500015013 | 0.204143002 |

| | | |
|---|---|---|
| 0.998754990 | 0.500042969 | 0.281698990 |
| 0.249936007 | 0.750015035 | 0.204143002 |
| 0.248754990 | 0.750042947 | 0.281698990 |
| 0.498754990 | 0.500042969 | 0.281698990 |
| 0.749935988 | 0.750015035 | 0.204143002 |
| 0.748754951 | 0.750042947 | 0.281698990 |
| 0.422493986 | 0.078377998 | 0.184014999 |
| 0.076277002 | 0.078399999 | 0.301724987 |
| 0.342130006 | 0.000077000 | 0.301073982 |
| 0.156433005 | -0.000011006 | 0.184658011 |
| 0.076187999 | 0.421669979 | 0.301739994 |
| 0.422448998 | 0.421651004 | 0.184032000 |
| 0.172493986 | 0.328377987 | 0.184014999 |
| 0.326276978 | 0.328399977 | 0.301724987 |
| 0.092129997 | 0.250077000 | 0.301073982 |
| 0.406432986 | 0.249988994 | 0.184658011 |
| 0.326187003 | 0.171670001 | 0.301739994 |
| 0.172448998 | 0.171651004 | 0.184032000 |
| 0.922493986 | 0.078377998 | 0.184014999 |
| 0.576277016 | 0.078399999 | 0.301724987 |
| 0.842129968 | 0.000077000 | 0.301073982 |
| 0.656433024 | -0.000011006 | 0.184658011 |
| 0.576187003 | 0.421669979 | 0.301739994 |
| 0.922448998 | 0.421651004 | 0.184032000 |
| 0.672494024 | 0.328377987 | 0.184014999 |
| 0.826277054 | 0.328399977 | 0.301724987 |
| 0.592130006 | 0.250077000 | 0.301073982 |
| 0.906432986 | 0.249988994 | 0.184658011 |
| 0.826187003 | 0.171670001 | 0.301739994 |
| 0.672448960 | 0.171651004 | 0.184032000 |
| 0.422493986 | 0.578378009 | 0.184014999 |
| 0.076277002 | 0.578400021 | 0.301724987 |
| 0.342130006 | 0.500077000 | 0.301073982 |

| | | |
|---|---|---|
| 0.156433005 | 0.499988994 | 0.184658011 |
| 0.076186998 | 0.921669979 | 0.301739994 |
| 0.422448998 | 0.921651048 | 0.184032000 |
| 0.172493986 | 0.828377987 | 0.184014999 |
| 0.326276978 | 0.828399999 | 0.301724987 |
| 0.092129997 | 0.750077022 | 0.301073982 |
| 0.406432986 | 0.749988972 | 0.184658011 |
| 0.326187003 | 0.671670001 | 0.301739994 |
| 0.172448998 | 0.671651026 | 0.184032000 |
| 0.922493986 | 0.578378009 | 0.184014999 |
| 0.576277016 | 0.578400021 | 0.301724987 |
| 0.842129968 | 0.500077000 | 0.301073982 |
| 0.656433024 | 0.499988994 | 0.184658011 |
| 0.576187003 | 0.921669979 | 0.301739994 |
| 0.922448998 | 0.921651048 | 0.184032000 |
| 0.672494024 | 0.828377987 | 0.184014999 |
| 0.826277054 | 0.828399999 | 0.301724987 |
| 0.592130006 | 0.750077022 | 0.301073982 |
| 0.906432986 | 0.749988972 | 0.184658011 |
| 0.826187994 | 0.671670001 | 0.301739994 |
| 0.672448960 | 0.671651026 | 0.184032000 |

**The atomic coordinates of the optimized computational models of CdS structure**

1.0

| | | |
|---|---|---|
| 11.6199998856 | 0.0000000000 | 0.0000000000 |
| 0.0000000000 | 8.2166004181 | 0.0000000000 |
| 0.0000000000 | 0.0000000000 | 23.2166004181 |

Cd S

20 20

Direct

| | | |
|---|---|---|
| 0.979447005 | 0.000000000 | 0.012849000 |
| 0.008833001 | 0.000000000 | 0.176937994 |
| 0.979431001 | -0.000000000 | 0.341064012 |
| 0.263431994 | 0.250000000 | 0.271210017 |
| 0.263458011 | 0.250000000 | 0.082714997 |
| 0.479447005 | 0.000000000 | 0.012849000 |
| 0.508832978 | 0.000000000 | 0.176937994 |
| 0.479431001 | -0.000000000 | 0.341064012 |
| 0.763432056 | 0.250000000 | 0.271210017 |
| 0.763457990 | 0.250000000 | 0.082714997 |
| 0.979447005 | 0.500000000 | 0.012849000 |
| 0.008833001 | 0.500000000 | 0.176937994 |
| 0.979431001 | 0.500000000 | 0.341064012 |
| 0.263431994 | 0.750000000 | 0.271210017 |
| 0.263458011 | 0.750000000 | 0.082714997 |
| 0.479447005 | 0.500000000 | 0.012849000 |
| 0.508832978 | 0.500000000 | 0.176937994 |
| 0.479431001 | 0.500000000 | 0.341064012 |
| 0.763432056 | 0.750000000 | 0.271210017 |
| 0.763457990 | 0.750000000 | 0.082714997 |
| 0.132255999 | -0.000000000 | 0.087270999 |
| 0.132227007 | -0.000000000 | 0.266626000 |
| 0.377065019 | 0.250000000 | 0.176963996 |
| 0.369422000 | 0.250000000 | 0.987345000 |
| 0.369428976 | 0.250000000 | 0.366576995 |

| | | |
|---|---|---|
| 0.632255958 | -0.000000000 | 0.087270999 |
| 0.632226986 | -0.000000000 | 0.266626000 |
| 0.877064978 | 0.250000000 | 0.176963996 |
| 0.869422041 | 0.250000000 | 0.987345000 |
| 0.869429017 | 0.250000000 | 0.366576995 |
| 0.132255999 | 0.500000000 | 0.087270999 |
| 0.132227007 | 0.500000000 | 0.266626000 |
| 0.377065019 | 0.750000000 | 0.176963996 |
| 0.369422000 | 0.750000000 | 0.987345986 |
| 0.369428976 | 0.750000000 | 0.366576995 |
| 0.632255958 | 0.500000000 | 0.087270999 |
| 0.632228012 | 0.500000000 | 0.266626000 |
| 0.877064978 | 0.750000000 | 0.176963996 |
| 0.869422041 | 0.750000000 | 0.987345986 |
| 0.869429017 | 0.750000000 | 0.366576995 |

**The atomic coordinates of the optimized computational models of CdPS3/CdS structure**

1.0

| | | |
|---|---|---|
| 12.0635995865 | 0.0000000000 | 0.0000000000 |
| 0.0000000000 | 21.1016998291 | 0.0000000000 |
| 0.0000000000 | 0.0000000000 | 50.0000000000 |

| | | |
|---|---|---|
| Cd | P | S |
| 46 | 16 | 78 |

Direct

| | | |
|---|---|---|
| 0.258840002 | 0.427080027 | 0.141660004 |
| 0.258830002 | 0.086260000 | 0.141629992 |
| 0.036420000 | 0.170440005 | 0.141660004 |
| 0.036410000 | 0.342900010 | 0.141629992 |
| 0.777220009 | 0.427080027 | 0.141660004 |
| 0.777220009 | 0.086260000 | 0.141629992 |
| 0.518029976 | 0.170440005 | 0.141660004 |
| 0.518029976 | 0.342900010 | 0.141629992 |
| 0.258840002 | 0.940349991 | 0.141660004 |
| 0.258830002 | 0.599539986 | 0.141629992 |
| 0.036420000 | 0.683720003 | 0.141660004 |
| 0.036410000 | 0.856180053 | 0.141629992 |
| 0.777220009 | 0.940349991 | 0.141660004 |
| 0.777220009 | 0.599539986 | 0.141629992 |
| 0.518029976 | 0.683720003 | 0.141660004 |
| 0.518029976 | 0.856180053 | 0.141629992 |
| 0.000860000 | 0.996730019 | 0.233940010 |
| 0.000860000 | 0.996730019 | 0.316100006 |
| 0.241669994 | 0.094080002 | 0.275020008 |
| 0.482480001 | 0.996730019 | 0.233940010 |
| 0.482480001 | 0.996730019 | 0.316100006 |
| 0.723280047 | 0.094080002 | 0.275020008 |
| 0.000860000 | 0.191420009 | 0.233940010 |
| 0.000860000 | 0.191420009 | 0.316100006 |
| 0.241669994 | 0.288769998 | 0.275020008 |

| | | |
|---|---|---|
| 0.482480001 | 0.191420009 | 0.233940010 |
| 0.482480001 | 0.191420009 | 0.316100006 |
| 0.723280047 | 0.288769998 | 0.275020008 |
| 0.000860000 | 0.386109998 | 0.233940010 |
| 0.000860000 | 0.386109998 | 0.316100006 |
| 0.241669994 | 0.483460010 | 0.275020008 |
| 0.482480001 | 0.386109998 | 0.233940010 |
| 0.482480001 | 0.386109998 | 0.316100006 |
| 0.723280047 | 0.483460010 | 0.275020008 |
| 0.000860000 | 0.580799988 | 0.233940010 |
| 0.000860000 | 0.580799988 | 0.316100006 |
| 0.241669994 | 0.678149999 | 0.275020008 |
| 0.482480001 | 0.580799988 | 0.233940010 |
| 0.482480001 | 0.580799988 | 0.316100006 |
| 0.723280047 | 0.678149999 | 0.275020008 |
| 0.000860000 | 0.775489977 | 0.233940010 |
| 0.000860000 | 0.775489977 | 0.316100006 |
| 0.241669994 | 0.872839989 | 0.275020008 |
| 0.482480001 | 0.775489977 | 0.233940010 |
| 0.482480001 | 0.775489977 | 0.316100006 |
| 0.723280047 | 0.872839989 | 0.275020008 |
| 0.518320024 | 0.000020000 | 0.119099998 |
| 0.035480000 | 0.000040000 | 0.164340000 |
| 0.259129992 | 0.256650009 | 0.119099998 |
| 0.257899992 | 0.256680018 | 0.164340000 |
| 0.036710002 | 0.000020000 | 0.119099998 |
| 0.517099986 | 0.000040000 | 0.164340000 |
| 0.777509978 | 0.256650009 | 0.119099998 |
| 0.776290019 | 0.256680018 | 0.164340000 |
| 0.518320024 | 0.513289985 | 0.119099998 |
| 0.035480000 | 0.513320040 | 0.164340000 |
| 0.259129992 | 0.769930007 | 0.119099998 |
| 0.257899992 | 0.769960016 | 0.164340000 |

| | | |
|---|---|---|
| 0.036710002 | 0.513289985 | 0.119099998 |
| 0.517099986 | 0.513320040 | 0.164340000 |
| 0.777509978 | 0.769930007 | 0.119099998 |
| 0.776290019 | 0.769960016 | 0.164340000 |
| 0.438030022 | 0.080459998 | 0.107349997 |
| 0.079079999 | 0.080480002 | 0.176019993 |
| 0.354710002 | 0.000080000 | 0.175640011 |
| 0.162190007 | -0.000010033 | 0.107729998 |
| 0.078989996 | 0.432869990 | 0.176029987 |
| 0.437979981 | 0.432850015 | 0.107360001 |
| 0.178839989 | 0.337099991 | 0.107349997 |
| 0.338279998 | 0.337119989 | 0.176019993 |
| 0.095519999 | 0.256720015 | 0.175640011 |
| 0.421380010 | 0.256630011 | 0.107729998 |
| 0.338179995 | 0.176230003 | 0.176029987 |
| 0.178790007 | 0.176210004 | 0.107360001 |
| 0.956419969 | 0.080459998 | 0.107349997 |
| 0.597469991 | 0.080480002 | 0.176019993 |
| 0.873099950 | 0.000080000 | 0.175640011 |
| 0.680570004 | -0.000010033 | 0.107729998 |
| 0.597370028 | 0.432869990 | 0.176029987 |
| 0.956370007 | 0.432850015 | 0.107360001 |
| 0.697220015 | 0.337099991 | 0.107349997 |
| 0.856659985 | 0.337119989 | 0.176019993 |
| 0.613899996 | 0.256720015 | 0.175640011 |
| 0.939759997 | 0.256630011 | 0.107729998 |
| 0.856570021 | 0.176230003 | 0.176029987 |
| 0.697179935 | 0.176210004 | 0.107360001 |
| 0.438030022 | 0.593729956 | 0.107349997 |
| 0.079079999 | 0.593760011 | 0.176019993 |
| 0.354710002 | 0.513360036 | 0.175640011 |
| 0.162190007 | 0.513260022 | 0.107729998 |
| 0.078989996 | 0.946140000 | 0.176029987 |

| | | |
|---|---|---|
| 0.437979981 | 0.946120024 | 0.107360001 |
| 0.178839989 | 0.850369978 | 0.107349997 |
| 0.338279998 | 0.850390044 | 0.176019993 |
| 0.095519999 | 0.769990025 | 0.175640011 |
| 0.421380010 | 0.769899998 | 0.107729998 |
| 0.338179995 | 0.689499979 | 0.176029987 |
| 0.178790007 | 0.689480003 | 0.107360001 |
| 0.956419969 | 0.593729956 | 0.107349997 |
| 0.597469991 | 0.593760011 | 0.176019993 |
| 0.873099950 | 0.513350003 | 0.175640011 |
| 0.680570004 | 0.513260022 | 0.107729998 |
| 0.597370028 | 0.946140000 | 0.176029987 |
| 0.956370007 | 0.946120024 | 0.107360001 |
| 0.697220015 | 0.850369978 | 0.107349997 |
| 0.856659985 | 0.850390044 | 0.176019993 |
| 0.613899996 | 0.769990025 | 0.175640011 |
| 0.939759997 | 0.769899998 | 0.107729998 |
| 0.856570021 | 0.689499979 | 0.176029987 |
| 0.697179935 | 0.689480003 | 0.107360001 |
| 0.121270001 | 0.996730019 | 0.275020008 |
| 0.362069997 | 0.094080002 | 0.316100006 |
| 0.362069997 | 0.094080002 | 0.233940010 |
| 0.602880004 | 0.996730019 | 0.275020008 |
| 0.843690010 | 0.094080002 | 0.316100006 |
| 0.843690010 | 0.094080002 | 0.233940010 |
| 0.121270001 | 0.191420009 | 0.275020008 |
| 0.362069997 | 0.288769998 | 0.316100006 |
| 0.362069997 | 0.288769998 | 0.233940010 |
| 0.602880004 | 0.191420009 | 0.275020008 |
| 0.843690010 | 0.288769998 | 0.316100006 |
| 0.843690010 | 0.288769998 | 0.233940010 |
| 0.121270001 | 0.386109998 | 0.275020008 |
| 0.362069997 | 0.483460010 | 0.316100006 |

| | | |
|---|---|---|
| 0.362069997 | 0.483460010 | 0.233940010 |
| 0.602880004 | 0.386109998 | 0.275020008 |
| 0.843690010 | 0.483460010 | 0.316100006 |
| 0.843690010 | 0.483460010 | 0.233940010 |
| 0.121270001 | 0.580799988 | 0.275020008 |
| 0.362069997 | 0.678149999 | 0.316100006 |
| 0.362069997 | 0.678149999 | 0.233940010 |
| 0.602880004 | 0.580799988 | 0.275020008 |
| 0.843690010 | 0.678149999 | 0.316100006 |
| 0.843690010 | 0.678149999 | 0.233940010 |
| 0.121270001 | 0.775489977 | 0.275020008 |
| 0.362069997 | 0.872839989 | 0.316100006 |
| 0.362069997 | 0.872839989 | 0.233940010 |
| 0.602880004 | 0.775489977 | 0.275020008 |
| 0.843690010 | 0.872839989 | 0.316100006 |
| 0.843690010 | 0.872839989 | 0.233940010 |
